# Fast Constrained Spectral Clustering and Cluster Ensemble with Random Projection

**DOI:** 10.1155/2017/2658707

**Published:** 2017-09-25

**Authors:** Wenfen Liu, Mao Ye, Jianghong Wei, Xuexian Hu

**Affiliations:** ^1^Guangxi Key Laboratory of Cryptogpraphy and Information Security, School of Computer Science and Information Security, Guilin University of Electronic Technology, Guilin 541004, China; ^2^State Key Laboratory of Networking and Switching Technology, Beijing University of Posts and Telecommunications, Beijing 100876, China; ^3^State Key Laboratory of Mathematical Engineering and Advanced Computing, Zhengzhou 450002, China; ^4^National University of Defense Technology, Nanjing 210012, China

## Abstract

Constrained spectral clustering (CSC) method can greatly improve the clustering accuracy with the incorporation of constraint information into spectral clustering and thus has been paid academic attention widely. In this paper, we propose a fast CSC algorithm via encoding landmark-based graph construction into a new CSC model and applying random sampling to decrease the data size after spectral embedding. Compared with the original model, the new algorithm has the similar results with the increase of its model size asymptotically; compared with the most efficient CSC algorithm known, the new algorithm runs faster and has a wider range of suitable data sets. Meanwhile, a scalable semisupervised cluster ensemble algorithm is also proposed via the combination of our fast CSC algorithm and dimensionality reduction with random projection in the process of spectral ensemble clustering. We demonstrate by presenting theoretical analysis and empirical results that the new cluster ensemble algorithm has advantages in terms of efficiency and effectiveness. Furthermore, the approximate preservation of random projection in clustering accuracy proved in the stage of consensus clustering is also suitable for the weighted *k*-means clustering and thus gives the theoretical guarantee to this special kind of *k*-means clustering where each point has its corresponding weight.

## 1. Introduction

With the arrival of the big data era, data has become an important asset. How to analyse the large scale data efficiently is becoming a big challenge [1, 2]. As an underlying method for data analysis, clustering can partition a data set into several subsets according to the similarities of points [3], and it has become a basic tool for image analysis [4, 5], community detection [6, 7], disease diagnosis [8], and so on. Therefore, more and more attention has been paid to the design of efficient and effective clustering algorithms.

Constrained clustering can improve the accuracy of the clustering result via encoding constraint information into unsupervised clustering. As an important area of clustering, many constrained clustering algorithms [9–17] have been proposed. Since spectral clustering often has high clustering accuracy and the suitability for a wide range of geometries [18, 19], constrained spectral clustering (CSC) [11–17] can usually have better performance than other constrained clustering algorithms. However, the *O*(*n*^2^) space complexity and *O*(*n*^3^) time complexity of many CSC algorithms [11–15] restrict their applications over large scale data sets, where *n* is the number of data points. The most efficient CSC algorithm known is SCACS algorithm [16], which reduces the space and time complexities to be linear with *n* through incorporating the landmark-based graph construction [20, 21] with the constrained normalized cuts problem [15]. What is needed to be noticed is that the constrained normalized cuts problem [15] makes SCACS algorithm solve the generalized eigenvector problem twice. In 2016, Cucuringu et al. [17] proposed a new CSC algorithm with better accuracy and shorter running time empirically than constrained normalized cuts problem. Taking a new encoding technique of constraint information, the new CSC model just needs the computation of eigenvectors once.

By means of integrating many basic partitions into a unified partition, ensemble clustering has many excellent properties such as the improvement of clustering quality, the robustness and stability of clustering results, the handling of noise, the reuse of knowledge [3], and the suitability to multisource and heterogeneous data [22]. Researchers have proposed many ensemble clustering algorithms [22–29]. Since there are different notations in different literatures, we call the integration of basic partitions as ensemble clustering or consensus clustering and call the union of the stages of basic clustering and ensemble clustering as cluster ensemble in the following. Among different ensemble clustering methods, the method based on coassociation matrix has become a landmark [22]. Specifically, the coassociation matrix is constructed to represent the similarities of pairs of points from the basic partitions and the final partition result is computed via the graph partition method on the matrix. Thus, this kind of method suffers from the high space and time complexity. Recently, Liu et al. [22] transformed spectral clustering on coassociation matrix to weighted *k*-means clustering over specific binary matrix equivalently, which decreased the space and time complexities vastly. However, when the number of basic partitions or clusters is large, the corresponding binary matrix will be high dimensional.

As the seminal work, Johnson and Lindenstrauss [30] pointed out that the random projection produced by random orthogonal matrix could preserve the pairwise distances of data sets approximately with reduced dimensions. Subsequently, a lot of researches constructed more matrices with the above properties: random Gaussian matrix [31], random sign matrix [32], random matrix based on randomized Hadamard transform [33], random matrix based on block random hashing [34], and so on. In addition, dimensionality reduction with random projection has also been widely applied to data mining methods such as classification [35], clustering [36–38], and anomaly detection [39]. In terms of object function, there are several works [36–38] to prove that random projection can maintain the accuracy of *k*-means clustering approximately. Since its objective function is different from that of *k*-means clustering, the theoretical analysis of the influence of random projection on weighted *k*-means clustering is still scarce.

*Our Contribution*. In this paper, our contributions can be divided into three parts: the first part is the proposition of a fast CSC algorithm which is suitable for a wide range of data sets; the second part is the analysis of the effect of random projection on the spectral ensemble clustering; the third part is the proposition of a scalable semisupervised cluster ensemble algorithm. More specifically, the contributions are as follows:

- We propose a fast CSC algorithm whose space and time complexities are linear with the size of a data set: we compress the size of the original model proposed by Cucuringu et al. [17] by the encoding of landmark-based graph construction and improve the efficiency further via random sampling in the process of *k*-means clustering. Besides, we prove that the new CSC algorithm will have the comparable clustering result of the original model asymptotically. Experimental results show that the new algorithm not only can utilize the constraint information effectively, but also costs less running time and fits a wider range of data sets compared to the state of the art SCACS method.
- With respect to the difference of objective function caused by random projection, we give a detailed proof that random projection can keep the clustering quality of spectral ensemble clustering within a small factor. Based on this theoretical analysis, we design a spectral ensemble clustering algorithm with reduced dimensions caused by sparse random projection. Experiments over different data sets also verify the correctness of our theoretical results. Moreover, since the theoretical analysis is also suitable for the ordinary weighted *k*-means clustering, the influence of random projection on weighted *k*-means clustering is also obtained.
- We propose a scalable semisupervised cluster ensemble algorithm through the combination of the fast CSC algorithm and spectral ensemble clustering algorithm with random projection. The efficiency and effectiveness of the new cluster ensemble algorithm are also demonstrated theoretically and empirically.

The remainder of our paper is organized as follows. In Section 2, we introduce the CSC model of Cucuringu et al. [17], landmark-based graph construction, and two related components in our cluster ensemble algorithm: spectral ensemble clustering and random projection. In Section 3, we present our fast CSC algorithm and give its asymptotic property. Then, the algorithm formulation and theoretical analysis of spectral ensemble clustering with random projection are displayed in Section 4. In Section 5, we show the experiment results of our algorithms. Finally, we draw the conclusions of the article and put forward the future directions in Section 6.

## 2. Preliminaries

In this section, we present the CSC algorithm proposed by Cucuringu et al. [17] and introduce landmark-based graph construction [20, 21] which will be applied to our fast CSC algorithm. In addition, we also introduce spectral ensemble clustering algorithm [22] and sparse random projection [34] which can be used to speed up the spectral ensemble clustering.

### 2.1. Constrained Spectral Clustering

Here, we first introduce the notion of undirected graph which is very important in constrained spectral clustering and then show the CSC model proposed by Cucuringu et al. [17].

Let *G* = (*V*, *E*, *W*) be an undirected graph, where *V* = {*v*_1_, *v*_2_,…, *v*_*n*_} is the vertex set, *E* is the edge set, and *W* is the weight set with respect to the edges. *w*_*ij*_ = *w*_*ji*_ is specially the nonnegative weight of the edge between the vertices *v*_*i*_ and *v*_*j*_, indicating the level of “affinity” between *v*_*i*_ and *v*_*j*_. If *w*_*ij*_ = 0, there is no edge between the vertices *v*_*i*_ and *v*_*j*_. We denote **L**_*G*_ = **D** − **W** as the Laplacian matrix of *G*, where the diagonal entry of diagonal matrix **D** is **D**(*i*, *i*) = ∑_*j*≠*i*_*w*_*ij*_; **W** is an adjacency matrix with **W**(*i*, *j*) = **W**(*j*, *i*) = *w*_*ij*_.

The constrained spectral clustering has three undirected graphs: one data graph *G*_*D*_ and two knowledge graphs *G*_ML_ and *G*_CL_. In data graph *G*_*D*_ = (*V*, *E*_*D*_, *W*_*D*_), each weight indicates the similarity level of vertices in the corresponding edge. The “must link” (ML) graph *G*_ML_ = (*V*, *E*_ML_, *W*_ML_) gives the “must link” information of vertices: each edge in *G*_ML_ indicates that the corresponding vertices should be in the same group and the level of “must link” belief is described by the weight. The “cannot-link” (CL) graph *G*_CL_ = (*V*, *E*_CL_, *W*_CL_) has analogous components to *G*_ML_. The values of weights in the two knowledge graphs are both nonnegative and set according to the constraint information such as prior knowledge. For example, assuming that the range of value of weight is set from 0 to 1, if we have known that points *v*_1_, *v*_2_ are in the same group, their corresponding weight *w*_ML,12_ = 1. If we only have 40% confidence in the constraint information that the two points are in the same group, the weight *w*_ML,12_ = 0.4, and if we have no constraint information about these two points, *w*_ML,12_ = *w*_CL,12_ = 0.

Viewing pairwise similarities of vertices as the implicit ML constraints declaration, Cucuringu et al. [17] defined a generalized ML graph ${\tilde{G}}_{D}\left[\alpha \right]=\left(V,E_{D}\cup E_{ML},W_{D}+\alpha *W_{ML}\right)$ where *α* is the level of trust for ML constrains. Let *k* be the number of clusters and **x**_*C*_*i*__ be the indicator vector of cluster *C*_*i*_ such that **x**_*C*_*i*__(*j*) = 1 if the *j*th data point belongs to cluster *C*_*i*_ and **x**_*C*_*i*__(*j*) = 0 otherwise. In order to violate as few ML constraints as possible and meet as many CL constraints as possible, the constrained *k* way cuts problem [17] can be described as

(1) arg⁡minxC1,xC2,…,xCk maxx∈xC1,xC2,…,xCk⁡xTLG~DxxTLGCLxs.t. ∑i=1kxCi=1n,xCi∈0,1n.

To solve the problem in (1) approximately, Cucuringu et al. [17] relaxed the condition “**x**_*i*_ ∈ {0,1}^*n*^, ∑_*i*=1_^*k*^**x**_*i*_ = {1}^*n*^” to be the real vectors. Thus, the solution vectors of the relaxed problem are the first *k* nontrivial generalized eigenvectors of the problem

(2) $$\begin{array}{c} L_{{\tilde{G}}_{D}}x=\lambda L_{G_{CL}}x. \end{array}$$

After getting the generalized eigenvectors, an additional embedding phase embeds the row vectors of eigenvectors matrix onto the *k*-dimensional sphere and gives the theoretical guarantees of clustering results. The detailed embedding procedures can be accessed in [17]. However, the construction cost and storage cost of data graphs for large scale data sets are both huge (*O*(*n*^2^)). What is more, if the number of iterations in the process of *k*-means clustering on the embedded eigenvectors matrix is great, the process will also be time-consuming over large scale data sets.

### 2.2. Landmark-Based Graph Construction

Based on sparse coding theory [40], the landmark-based graph construction [20, 21] scales linearly with the number of data points and can suit large scale data sets very well.

Let data set be **A** ∈ *ℝ*^*n*×*d*^ and the row vector **a**_*i*_ of **A** be data points; sparse coding problem is defined as follows:

(3) minU,Z AT−UZ2s.t. Z  is  sparse,

where each column vector of **U** ∈ *ℝ*^*d*×*p*^ is the basis vector, column vectors of **Z** ∈ *ℝ*^*p*×*n*^ are the representations of data points over **U** and *p* is the number of basis vectors. To avoid the high time complexity of solving sparse coding problem, landmark-based graph construction just samples points randomly from input data **A** as basis vectors. In the process of computing **Z**, if **u**_*j*_ is among the *r* nearest basis vectors of data points **a**_*i*_, **Z**(*j*, *i*) can be computed as

(4) $$\begin{array}{c} Z\left(\;j,i\;\right)=\frac{K_{\sigma }\left(a_{i},u_{j}\right)}{\sum_{j^{\prime }\in U\left(i,r\right)}K_{\sigma }\left(a_{i},u_{j^{\prime }}\right)}, \end{array}$$

where *U*(*i*, *r*) is the indices set of the *r* nearest basis vectors of **a**_*i*_ and *K*_*σ*_(·) is Gaussian kernel function with bandwidth *σ*; otherwise **Z**(*j*, *i*) = 0.

After obtaining the sparse representation **Z** ∈ *ℝ*^*p*×*n*^, graph affinity matrix is constructed as follows:

(5) $$\begin{array}{c} W={\hat{Z}}^{T}\hat{Z}, \end{array}$$

where $\hat{Z}=D^{-1/2}Z$ and **D** is a diagonal matrix with diagonal entry **D**(*i*, *i*) = ∑_*j*_**Z**(*i*, *j*). Since Chen and Cai [20, 21] have pointed out that **W** was automatically normalized, the normalized graph Laplacian matrix for **A** is $I-{\hat{Z}}^{T}\hat{Z}$. Considering *p* ≪ *n*, the *O*(*npd*) time of computing $\hat{Z}$ is much less than the *O*(*n*^2^*d*) time of the nearest neighbors graph construction.

### 2.3. Spectral Ensemble Clustering

To gain the unified results from different basic partitions, spectral ensemble clustering applies spectral clustering to the coassociation matrix [24] derived from basic partitions. In 2015, Liu et al. [22] transformed spectral ensemble clustering into weighted *k*means clustering over specific binary matrix. This transformation decreased the time and space complexities effectively and our new ensemble clustering method is based on this nice transformation.

Given *g* basic clustering results Π = {*π*_1_, *π*_2_,…, *π*_*g*_} of data set **A** ∈ *ℝ*^*n*×*d*^; the coassociation matrix **C** is constructed in the following way:

(6) $$\begin{array}{c} C\left(\;j,k\;\right)=\overset{g}{\underset{i=1}{\sum }}\eta \left(\;\pi_{i}\left(\;a_{j}\;\right),\pi_{i}\left(\;a_{k}\;\right)\;\right), \end{array}$$

where *π*_*i*_(**a**_*j*_) is the label of **a**_*j*_ in the *i*th clustering result *π*_*i*_, and

(7) $$\begin{array}{c} \eta \left(\;a,b\;\right)=\left\{\begin{array}{cc} 1, & if\;\;a=b\\ 0, & if\;\;a\neq b. \end{array}\right. \end{array}$$

Viewing this coassociation matrix as adjacency matrix, spectral ensemble clustering uses spectral clustering to get final clustering result. In the process of the transformation from spectral clustering to weighted *k*-means clustering, binary matrix **B** = {**b**(**a**)} [22] is built as follows:

(8) $$\begin{array}{c} b\left(\;a\;\right)=\left[\;b{\left(\;a\;\right)}_{1},\ldots ,b{\left(\;a\;\right)}_{g}\;\right], \end{array}$$

where **b**(**a**)_*i*_ = [*b*(**a**)_*i*1_,…, *b*(**a**)_*ik*_*i*__], *b*(**a**)_*ij*_ = 1 if *π*_*i*_(**a**) = *j*, and *b*(**a**)_*ij*_ = 0 otherwise; “[]” indicates a row vector. The following lemma [22] presents the connection between spectral ensemble clustering and weighted *k*-means clustering.

**Lemma 1 (see [22]).**

Given a basic partitions set Π, let the corresponding coassociation matrix be **C**, the diagonal matrix whose diagonal elements are sums of rows of **C** be **D**1, and the diagonal element set of **D**1 be {*w*_**b**(**a**)_}. Then normalized cuts spectral clustering on coassociation matrix **C** has equivalent objective function to weighted *k*-means clustering on data sets {**b**(**a**)/*w*_**b**(**a**)_} with weight set {*w*_**b**(**a**)_}.

Through Lemma 1, the space and time complexities of spectral ensemble clustering can be decreased dramatically. However, when the number of basic partitions and cluster number are large, the binary matrix **B** will be a high dimensional data set, resulting in long running time for weighted *k*-means clustering.

### 2.4. Random Projection

Recently, random projection has become a common technique of dimensionality reduction [36–39, 41]. Random projection often has low computing complexity and can preserve the structure of original data approximately. In this paper, we use the sparse random projection proposed by Kane and Nelson [34]. When most of the elements of data are zero, the sparse random projection can utilize the sparsity of data effectively and speed up the process of dimensionality reduction.

**Lemma 2 (see [34]).**

For any 0 < *δ*, *ε* < 1/2, *d* > 0, there exists an *d* × (*av*) sparse random matrix **R**, where *a* = Θ(*ε*^−1^log(1/*δ*)) and *v* = Θ(*ε*^−1^), such that for any fixed **x** ∈ *ℝ*^*d*^

(9) $$\begin{array}{c} Pr\left\{\;\left(\;1-\varepsilon \;\right){\left\|\;x\;\right\|}_{2}^{2}\leq {\left\|\;R^{T}x\;\right\|}_{2}^{2}\leq \left(\;1+\varepsilon \;\right){\left\|\;x\;\right\|}_{2}^{2}\;\right\}>1-\delta . \end{array}$$

And the random matrix **R** can be constructed as follows:

(10) $$\begin{array}{c} R^{T}=\left[\begin{array}{c} \sqrt{\frac{1}{a}}\cdot \Phi_{1}\cdot D_{1}\\ \vdots \\ \sqrt{\frac{1}{a}}\cdot \Phi_{a}\cdot D_{a} \end{array}\right], \end{array}$$

where matrix Φ_*l*_ (*l* ∈ [1, *a*]) is a *v* × *d* sparse matrix with nonzero elements Φ(*h*(*i*), *i*) = 1, *h* : {1,…, *d*} → {1,…, *v*} is a random hashing such that Pr{*h*(*i*) = *j*} = 1/*v* for *i* ∈ {1,…, *d*}, *j* ∈ {1,…, *v*}, and matrix **D**_*l*_ is a *d* × *d* diagonal matrix with Pr{**D**_*l*_(*i*, *i*) = ±1} = 0.5.

The number of nonzero (nnz) elements of sparse random matrix **R** is *ad*, and the time complexity of **AR** is nnz(**A**)*a*.  Lemma 2 implies that the sparse random projection can preserve the length of data points approximately. Thus, for *n* data points, since there are *n*(*n* − 1)/2 pairwise distances, we can conclude that the pairwise distances squares can be preserved within a factor of 1 ± *ε* with *a* = Θ(2*ε*^−1^log⁡(*n*/*δ*)).

## 3. Fast Constrained Spectral Clustering Framework

In this section, we introduce our fast CSC framework for large scale data sets. Inspired by [20, 21], we also try to compute the sparse representation $\hat{Z}$ and obtain the approximate adjacency matrix $W={\hat{Z}}^{T}\hat{Z}$, where $\hat{Z}\in {\mathbb{R}}^{p\times n}$, and *p* ≪ *n*. Then, our fast framework decreases the size of graph Laplacian through the above approximate graph reconstruction. At last, we analyse the asymptotic property of our new CSC algorithm.

### 3.1. Framework Formulation

To get the generalized eigenvector **x** approximately, we can let $x={\hat{Z}}^{T}y$, where $\hat{Z}\in {\mathbb{R}}^{p\times n}$ is the sparse representation in (5) and **y** ∈ *ℝ*^*p*^. Thus, bringing the **x** back to (1) can decrease the size of problem apparently if *p* ≪ *n*.

Specifically, we use **Q** to denote constraint matrix, where **Q**(*i*, *j*) = 1 if edge (**v**_*i*_, **v**_*j*_) ∈ *E*_ML_, **Q**(*i*, *j*) = −1 if edge (**v**_*i*_, **v**_*j*_) ∈ *E*_CL_, and **Q**(*i*, *j*) = 0 otherwise. Let adjacency matrix be computed approximately by $W={\hat{Z}}^{T}\hat{Z}$. Next, bring $x={\hat{Z}}^{T}y$ into (1) and relax their solution over real vectors. Thus, we reformulate the original problem as the following problem.

**Problem 3 .**

One has

(11) arg⁡miny1,y2,…,yk maxy∈y1,y2,…,yk⁡yTZ^LG~DZ^TyyTZ^LGCLZ^Tys.t. yi∈Rpfor  any  i∈1,k.

To obtain shorthand notations, we denote $\hat{Z}L_{{\tilde{G}}_{D}}{\hat{Z}}^{T}$ by **L**_CGD_ and denote $\hat{Z}L_{G_{CL}}{\hat{Z}}^{T}$ by **L**_CCL_. Thus, the first *k* nontrivial generalized eigenvectors of the problem

(12) $$\begin{array}{c} L_{CGD}y=\lambda L_{CCL}y \end{array}$$

are the solution vectors of (11).

In order to speed up the *k*-means clustering on the embedded eigenvector matrix, we sample row vectors of eigenvectors matrix randomly and get *k* centers through *k*-means clustering over the selected row vectors. According to the distances between centers and row vectors, we can partition all the row vectors into different clusters. Cucuringu et al. [17] have pointed out that the specific embedding process after getting the generalized eigenvectors can concentrate the row vectors of eigenvector matrix onto the *k*-dimensional sphere and a simple partition algorithm such as *k*-means clustering can be applied to get the final clustering result. Since random sampling is a popular scalability method for *k*-means clustering [42], we will take it to improve the efficiency of the clustering on the row vectors of eigenvector matrix. The experimental results in Section 5 also show that random sampling has little influence on the clustering results and makes the algorithm more efficient than the original one.

Our fast CSC framework is shown in Algorithm 1. In our new algorithm, parameter *α* (in $L_{{\tilde{G}}_{D}}$ of Step (2)) stands for the trust level on constraint information. Since the *α* of the original problem (see (2)) has been taken to a constant in the previous work [17], we also set *α* as a constant.

The complexity analysis of Algorithm 1 is presented as follows. The time of computing $\hat{Z}$ is *O*(npd). In Step (2), the **L**_CGD_ is computed as follows:

(13) LCGD=Z^LG~DZ^T=Z^I−Z^TZ^+αLGMLZ^T=Z^Z^T−Z^Z^T2+αZ^LGMLZ^T.

Let the number of data points with constraint information be *c*; then the time cost for computing $\alpha \hat{Z}L_{G_{ML}}{\hat{Z}}^{T}$ is *O*(*p*^2^*c* + *pc*^2^). Hence, the time cost of Steps (1) and (2) is *O*(*p*^2^*n* + *p*^3^) + *O*(*p*^2^*c* + *pc*^2^) = *O*(*p*^2^*n* + *p*^3^ + *p*^2^*c* + *pc*^2^). Besides, the time complexity of Step (3) is *O*(*p*^3^), that of Step (4) is *O*(*kpn*), and that of Step (5) is *O*(*kn*). Thus, the time cost of the first 5 steps is *O*(*p*^2^*n*) considering *p*, *c* ≪ *n* and *k* ≪ *p*, *c*. Assuming the iteration numbers of *k*-means clustering are *l*, the time cost of Steps (6) and (7) is *O*((*ns*)*k*^2^*l* + *nk*^2^), which is much less than the time cost *O*(*nlk*^2^) of *k*-means clustering on $\hat{X}$ with (*ns*) ≪ *n*. Hence, the time complexity of our algorithm is

(14) $$\begin{array}{c} O\left(\;np^{2}+nk^{2}+npd\;\right). \end{array}$$

Since three matrices $\hat{Z}$, **L**_CGD_, and **L**_CCL_ are stored, the memory complexity is

(15) $$\begin{array}{c} O\left(\;np+p^{2}\;\right). \end{array}$$

### 3.2. Asymptotic Property of the Framework

In this subsection, we show that the partition result of our fast CSC algorithm could be comparable to that of the original model [17] as *p* converges to *n*.

**Theorem 4 .**

Assuming the adjacency matrix **W** in the original model is full rank, the result of Step (4) in Algorithm 1 will converge to the generalized eigenvectors of (2) as *p* converges to *n*.

**Proof**

From the construction of sparse representation $\hat{Z}$, we can get that

(16) $$\begin{array}{c} \underset{p\to n}{lim}\hat{\;Z}=\hat{W}, \end{array}$$

where $\hat{W}$ is the normalized adjacency matrix. Equation (12) can be rewritten as

(17) $$\begin{array}{c} \hat{Z}\left[\;I-\hat{W}+\alpha L_{G_{ML}}\;\right]{\hat{Z}}^{T}y=\lambda \hat{Z}L_{G_{CL}}{\hat{Z}}^{T}y. \end{array}$$

Equally, we have that

(18) $$\begin{array}{c} \hat{Z}\left[\;I-\hat{W}+\alpha L_{G_{ML}}-\lambda L_{G_{CL}}\;\right]{\hat{Z}}^{T}y=0. \end{array}$$

Since the rank of $\hat{Z}$ will be equal to *n*, $\hat{Z}$ can be removed. Thus the equation will be

(19) $$\begin{array}{c} \left[\;I-\hat{W}+\alpha L_{G_{ML}}\;\right]{\hat{Z}}^{T}y=\lambda L_{G_{CL}}{\hat{Z}}^{T}y. \end{array}$$

This equation shows that ${\hat{Z}}^{T}y$ and *λ* in Step (4) of Algorithm 1 are indeed the eigenvector and eigenvalue of (2), respectively. Moreover, the number of eigenvectors of (19) will converge to *n* as *p* converges *n*. Hence Algorithm 2 could also get all the eigenvectors of (2) asymptotically.

Since the eigenvectors of our framework will converge to that of original CSC model [17] and the random sampling has little influence on the clustering result of embedded eigenvectors matrix, our new CSC algorithm will generate the partition result which is comparable to that of original framework. In addition, the reason why we give the assumption of Theorem 4 is that each row vector of adjacency matrix is the similarity representation of certain point over the whole data set, and those representations are often linearly independent. In the experiments, we have demonstrated this theory empirically on the 30 nearest neighbors adjacency matrices of three data sets.

## 4. Spectral Ensemble Clustering with Random Projection

In this section, we propose an improved spectral ensemble clustering algorithm with random projection. The new ensemble clustering not only improves the efficiency of spectral ensemble clustering algorithm designed by Liu et al. [22], but also can theoretically preserve the approximate clustering result.

### 4.1. Algorithm Formulation

In this subsection, we give the detailed procedure of our new spectral ensemble clustering algorithm. We denote the original spectral ensemble clustering [22] by SEC and our improved spectral ensemble clustering with random projection by SECRP.

From the description of Section 2.3, we can know that the SEC algorithm transforms the spectral clustering on the coassociation matrix into weighted *k*-means clustering on the specific binary matrix **B**. The dimension of binary matrix **B** is ∑_*i*=1_^*g*^*k*_*i*_, where *k*_*i*_ is the cluster number of basic partition *π*_*i*_. When the number of clusters and/or basic partitions is big, **B** is probably a high dimensional matrix on which the weighted *k*-means clustering runs slowly.

To avoid the high dimensions of **B**, we design an improved SEC algorithm with random projection for dimensionality reduction. The new algorithm SECRP is showed in Algorithm 2.

The complexity analysis of the new algorithm is as follows. Obviously, the running time of Steps (1) and (2) is very short (compared with that of Step (3)). The time of Step (3) is *O*(*nnz*(**B**)*a*) = *O*(*nga*), where *g* is the number of basic partitions; *nnz*() denotes the number of nonzero entries. Another common method of dimensionality reduction is singular value decomposition (SVD). The time of running SVD on binary matrix **B** is *O*((*d*′)^3^ + *n*(*d*′)^2^), and that of the product between eigenvectors and **B** is *O*(*nd*′*va*). Since *g* ≈ *d*′/*k*, random projection with sparse random matrix is a cost-effective method of dimensionality reduction. With respect to the weighted *k*-means clustering, dimensionality reduction of random projection can decrease the running time of each iteration from *O*(*nkd*′) to *O*(*nkva*).

As a basic module, Algorithm 2 can be combined with different basic partition methods to produce different cluster ensemble algorithms. Thus, taking Algorithm 1 as the basic partition algorithm for Algorithm 2 could generate an efficient constrained cluster ensemble method with high accuracy (both basic partitions and final clustering are spectral clustering). Moreover, the last two steps of Algorithm 2 are just weighted *k*-means clustering with sparse random projection, which is also suitable for any other applications of weighted *k*-means clustering.

### 4.2. Theoretical Analysis of New Ensemble Algorithm

In this subsection, we demonstrate that our new algorithm SECRP can maintain the clustering result of SEC approximately.

For the theoretical analysis, we give the formal definition of weighted *k*-means clustering problem with matrix notation:

**Definition 5 (weighted *K*-means clustering problem).**

Given an *n* points set **B** (each row is a data point), diagonal matrix **W**_**B**_ whose diagonal entries set {*w*_**b**_} is weights set and clusters number *k* find an *n* × *k* indicator matrix **X**_opt_ such that

(20) $$\begin{array}{c} X_{opt}=\arg\underset{X}{\min}\;{\left\|\;W_{B}^{1/2}\left(\;B-XX^{T}W_{B}B\;\right)\;\right\|}_{F}^{2}, \end{array}$$

where ‖·‖_*F*_^2^ denotes the square of Frobenius norm; **X** is selected from the set of all indicator matrices. An indicator matrix has one nonzero element on each row. Specifically, if the *i*th point belongs to the *j*th cluster, $X(i,j)=1/\sqrt{w_{Cj}}$, where *w*_*Cj*_ denotes the sum of weights points in cluster *C*_*j*_.

Since computing **X**_opt_ is an NP-hard problem, we focus on the approximate algorithm for weighted *k*-means clustering. The corresponding definition is as follows.

**Definition 6 (weighted *K*-means approximation algorithm).**

An algorithm is called the “*γ*-approximation” for weighted *k*-means clustering problem, if the algorithm takes **B**, *k*, and **W**_**B**_ as input and outputs an indicator matrix **X**_*γ*_ such that

(21) PrWB1/2B−XγXγTWBBF2≤γ minX⁡WB1/2B−XXTWBBF2≥1−δγ,

where *γ* is the approximation factor and *δ*_*γ*_ is the failure probability of the “*γ*-approximation” weighted *k*-means clustering algorithm.

Though there is the *γ*-approximation *k*-means clustering algorithm such as [43], it is unclear whether the *γ*-approximation weighted *k*-means clustering algorithm exists or not. To facilitate the proof of our theory, we assume that the approximation algorithm exists and utilize the definition of approximation algorithm in the process of proof. And we will take the weighted version of the classical *k*-means clustering algorithm [44] as the weighted *k*-means clustering to verify our theoretical results in the following experiments.

**Theorem 7 .**

Let *n* × *d*′ matrix **B**, weight set {*w*_**b**(**a**)_}, and cluster number *k* be the inputs of Algorithm 2. Let *ε* ∈ (0,1/3). Assuming that a *γ*-approximation weighted *k*-means clustering algorithm exists, then the output $X_{\hat{\gamma }}$ of Algorithm 2 satisfies with probability of at least 0.97 − *δ*_*γ*_:

(22) WB1/2B~−Xγ^Xγ^TWBB~F2≤1+1+εγWB1/2B~−XoptXoptTWBB~F2.

In the above, $\tilde{B}=W_{B}^{-1}B$ is the computing result of Step (2) in Algorithm 2; **X**_opt_ is the optimal solution of weighted *k*-means clustering on $\tilde{B}$.

This theorem reveals that random projection not only can be used to improve the efficiency of spectral ensemble clustering with lower dimensions, but also maintains its final result approximately.

In the following, we present a useful lemma which is needed in the proof of Theorem 7. The results of the lemma are based on the results of [36] and Lemma 2.

**Lemma 8 .**

Let $\tilde{B}$, **R**, **W**_**B**_, *k*, and *ε* be the same as those in Theorem 7; denote $W_{B}^{1/2}\tilde{B}$ by **H**, the product of top *k* singular vectors (left and right) and singular values of **H** by **H**_*k*_.

(1) (Lemma 5 of [36]) Let the SVD of **H**_*k*_ be **H**_*k*_ = **U**_*k*_Σ_*k*_**V**_*k*_^*T*^, where **U**_*k*_ and **V**_*k*_ are the left and right singular vector matrices; Σ_*k*_ is a diagonal matrix whose diagonal elements are the *k* singular values. With probability of at least 0.97,

(23) $$\begin{array}{c} H_{k}=HR{\left(\;V_{k}^{T}R\;\right)}^{†}V_{k}^{T}+E, \end{array}$$

where ( )^†^ is the pseudoinverse of matrix; **E** is an *n* × *d*′ matrix with ‖**E**‖_*F*_ ≤ 4*ε*‖**H** − **H**_*k*_‖_*F*_.

(2) (Lemma 4 of [36]) For any *n* × *d*′ matrix **G**, with probability of at least 0.99,

(24) $$\begin{array}{c} {\left\|\;GR\;\right\|}_{F}\leq \sqrt{1+\varepsilon }{\left\|\;G\;\right\|}_{F}. \end{array}$$

(3) (Combination of Lemmas 2 and 3 of [36]) With probability of at least 0.99,

(25) $$\begin{array}{c} {\left\|\;{\left(\;V_{k}^{T}R\;\right)}^{†}\;\right\|}_{2}\leq \frac{1}{1-\varepsilon }. \end{array}$$

These conclusions are all about the influences of random matrix **R** on the norms of different matrices, which are useful for bounding the norms of the matrices in Theorem 7. In the following proof of Theorem 7, we start by decomposing the term ${\left\|W_{B}^{1/2}(\tilde{B}-X_{\hat{\gamma }}X_{\hat{\gamma }}^{T}W_{B}\tilde{B})\right\|}_{F}^{2}$ in (22). Then, based on the influences of random matrix in Lemma 8, we manipulate the norms of the different terms in the decomposition result.

**Proof**

Using the notation of Lemma 8, (22) can be decomposed into

(26) WB1/2B~−Xγ^Xγ^TWBB~F2=I−WB1/2Xγ^Xγ^TWB1/2WB1/2B~F2=I−WB1/2Xγ^Xγ^TWB1/2HF2=I−WB1/2Xγ^Xγ^TWB1/2HkF2+I−WB1/2Xγ^Xγ^TWB1/2Hρ−kF2,

where **H**_*ρ*−*k*_ = **H** − **H**_*k*_. The last equation is based on the orthogonality of **H**_*k*_ and **H**_*ρ*−*k*_.

We first give the bound of the second term of (26). According to our definition of indicator matrix, $X_{\hat{\gamma }}^{T}W_{B}^{1/2}W_{B}^{1/2}X_{\hat{\gamma }}=I_{k}$. Thus, $I-W_{B}^{1/2}X_{\hat{\gamma }}X_{\hat{\gamma }}^{T}W_{B}^{1/2}$ is a projector matrix; namely, its *l*_2_ norm is 1. As a result, we get

(27) I−WB1/2Xγ^Xγ^TWB1/2Hρ−kF2≤Hρ−kF2≤I−WB1/2XoptXoptTWB1/2HF2,

where the second inequality is caused by the fact that rank(**W**_**B**_^1/2^**X**_opt_**X**_opt_^*T*^**W**_**B**_^1/2^) ≤ *k* and the optimality of SVD.

We next bound the first term of (26). From the first statement of Lemma 8, we get

(28) I−WB1/2Xγ^Xγ^TWB1/2HkF≤I−WB1/2Xγ^Xγ^TWB1/2HRVkTR†VkTF+EF≤I−WB1/2Xγ^Xγ^TWB1/2HRFVkTR†F+EF.

From Definition 6 and the meaning of **X**_opt_ of Theorem 7, we get

(29) I−WB1/2Xγ^Xγ^TWB1/2HRF≤γ minX⁡I−WB1/2XXTWB1/2HRF≤γI−WB1/2XoptXoptTWB1/2HRF.

Using the statement 2 of Lemma 8, (29) can be transformed to

(30) I−WB1/2Xγ^Xγ^TWB1/2HRF≤γ1+εI−WB1/2XoptXoptTWB1/2H.

Combining the statement 3 of Lemma 8 and (30), we get

(31) I−WB1/2Xγ^Xγ^TWB1/2HRFVkTR†F+EF≤γ1+ε·I−WB1/2XoptXoptTWB1/2HF·VkTR†F+EF≤γ1+ε1−εI−WB1/2XoptXoptTWB1/2HF+EF≤γ1+ε1−ε+4ε·I−WB1/2XoptXoptTWB1/2HF.

From (28) and (31), and rescaling *ε*, we can get

(32) I−WB1/2Xγ^Xγ^TWB1/2HkF≤γ1+εI−WB1/2XoptXoptTWB1/2HF.

Finally, combining (27) and (32) concludes the proof.

It is easy to check that the above theoretical analysis can be also applied to ordinary weighted *k*-means clustering, indicating that the method of dimensionality reduction with random projection can preserve the clustering quality of weighted *k* means clustering approximately. Furthermore, the integration of Theorems 4 and 7 means that the new semisupervised cluster ensemble method (combination of Algorithms 1 and 2) can have an encouraging clustering result.

## 5. Experiments

In this section, we present the experimental results of our new algorithms in Sections 3 and 4. We implemented all the related algorithms in Matlab and conducted our experiments on a Windows machine with the Intel Core 3.6 GHz processor and 16 GB of RAM.

### 5.1. Data Sets and Experimental Settings

In order to facilitate the comparison, we performed experiments on three data sets which can be achieved from public web sites (http://archive.ics.uci.edu/ml/), (http://www.cad.zju.edu.cn/home/dengcai/).  Table 1 summarizes their basic information.

The constraint information is generated from the real labels of data sets. In our experiments, we sample the labeled points randomly from data sets. The constraint matrix **Q** is constructed as

(33) $$\begin{array}{c} Q\left(\;i,j\;\right)=\left\{\begin{array}{cc} 1 & x_{i},x_{j}\;\;have\;\;the\;\;same\;\;label\\ -1 & x_{i},x_{j}\;\;have\;\;different\;\;labels\\ 0 & no\;\;constraint. \end{array}\right. \end{array}$$

The validation measures of the partition result used in our experiments are cluster accuracy (CA) [45] and normalized mutual information (NMI) [25]. The CA is computed as

(34) $$\begin{array}{c} CA=\overset{k}{\underset{i=1}{\sum }}\frac{\max\left({cluster}_{i}\;|\;label\right)}{n}, \end{array}$$

where *k* is the cluster number of clustering result, *n* is the number of data points, max⁡(cluster_*i*_∣label) is the maximum number of points with the same true label in the *i*th cluster. For computing the NMI, we construct two random variables *C* and *L* from the clustering result and true label, respectively. The probability distributions of random variables are the proportions of different clusters (or classes) over the whole data set. The NMI is computed as follows:

(35) NMI=MIC,LHC·HL=∑c,lnc,llog⁡n·nc,l/nc·nl∑cnclog⁡nc/n∑lnllog⁡nl/n,

where MI(*C*, *L*) denotes the mutual information of random variables *C* and *L*, *H*(·) denotes the entropy of a random variable, *n* is the number of data points, *n*_*c*,*l*_ is the number of points in both cluster *c* and class *l*, *n*_*c*_ is the points number of cluster *c*, and *n*_*l*_ is the points number of class *l*. The values of CA and NMI both vary from 0 and 1, and the higher value means better clustering solution.

### 5.2. Comparisons of Different Constrained Spectral Clustering

In this subsection, we compare our fast CSC (constrained spectral clustering) algorithm with other spectral clustering algorithms. Following is the list of information of different algorithms in comparison:

- *LSC-R* [20, 21]: the unsupervised spectral clustering baseline with landmark-based graph construction.
- *SCACS* [16]: the most efficient CSC algorithm known and be set as the CSC baseline over MNIST and CoverType data sets.
- *CCS* [17]: the original CSC model proposed in [17], set as the CSC baseline over LetterRec data set. (Since the constructions of the nearest neighbors graphs are both time-consuming on MNIST and CoverType data sets, we do not run CCS algorithm on these two data sets.)
- *CCS-L*: our improved CCS algorithm with landmark-based graph construction.
- *CCS-LS*: our improved CCS algorithm with landmark-based graph construction and random sampling.

In the process of the landmark-based graph construction, we fix the number of landmark points *p* = 500 and the number of nearest neighbors *r* = 3. The parameters in SCACS algorithm that we used are *β*_0_ = 0.1, which is the same as those in [16]. Since in the original model CCS [17] it has been pointed out that *α* could be a constant number and *α* was set to 5 in their implementation code, we also set *α* = 5 in CCS, CCS-L, and CCS-LS.

**Figure 1:**
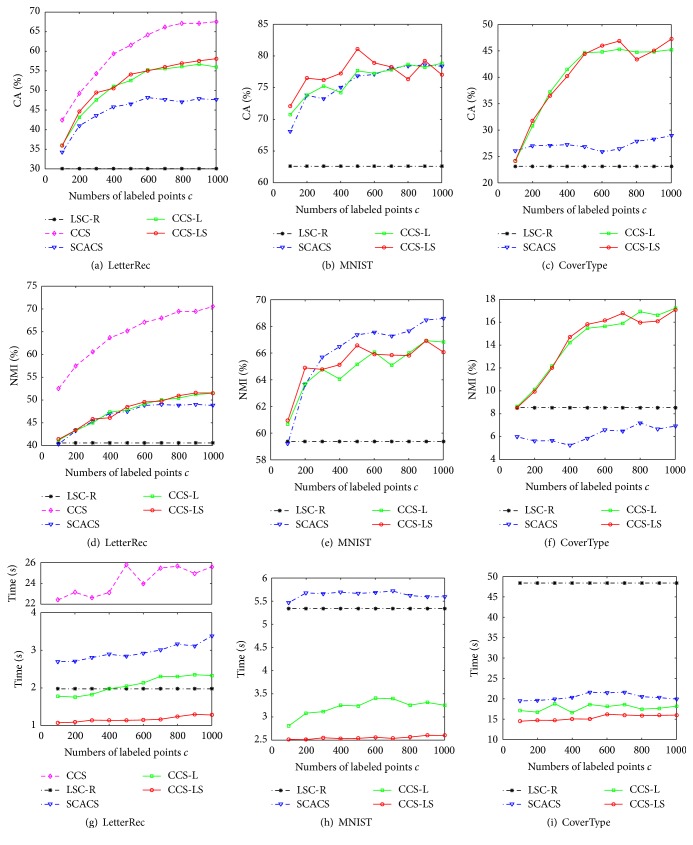
Performance of clustering algorithms with different constraint information.

First, we investigate the influence of the number of labeled points *c* on the performance of algorithms. We vary the value of *c* from 100 to 1000 with step size 100. For each value of *c*, we select the *c* labeled points randomly to produce constraint information and repeat 20 trials with different labeled points sets. The corresponding experimental results are presented in Figure 1. Figures 1(a), 1(b), and 1(c) are related to CA of clustering results, Figures 1(d), 1(e), and 1(f) are related to NMI, and Figures 1(g), 1(h), and 1(i) are related to running time. We can see that our algorithm CCS-LS outperforms LSC-R on all data sets and the values of CA and NMI increase with the growth of constraint information. Those indicate that our algorithm can employ the constraint information appropriately. Compared with SCACS, our algorithm has the similar performances on LetterRec and MNIST data sets and superior performances on CoverType data set, indicating that our algorithm adapts a wider range of geometries. Over the three data sets, the performances of CCS-LS are all close to CCS-L. What is more, our algorithm runs fastest among these algorithms.

**Figure 2:**
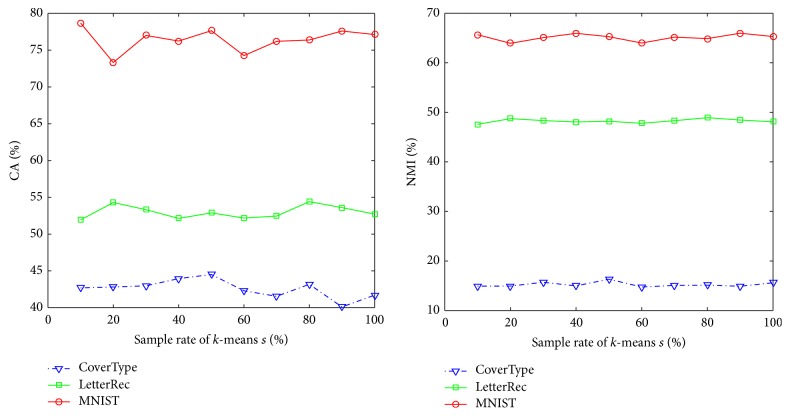
Influence of sample rates on proposed algorithms.

Next, we study the influence of random sampling (Step (5) of Algorithm 1) which can be seen in Figure 2. In the experiments, we fix *c* = 500 and change the sample rate from 0.1 to 1 by a step size 0.1. We still run 20 independent trials considering the randomness and compute the means of validity measures. We can see that the values of CA and NMI vary slightly along with the growth of sample rate, verifying the feasibility of random sampling.

### 5.3. Performance of the Spectral Ensemble Clustering with Random Projection

Since cluster ensemble consists of two parts: basic partition clustering and ensemble clustering, we below combine different basic partition clustering algorithms and different ensemble clustering algorithms to get different cluster ensemble algorithms. Thus, the performance of new ensemble clustering algorithm (Algorithm 2) and new cluster ensemble algorithm (combination of Algorithms 1 and 2) can both be manifested. Following is the list of information of different cluster ensemble algorithms in comparison:

- *CK-SE*: the basic partition clustering algorithm “CK” is the constrained *k*-means clustering algorithm [9], and the ensemble clustering algorithm “SE” is the spectral ensemble clustering (SEC) algorithm [22].
- *SCACS-SE*: the basic partition clustering algorithm is SCACS [16] in Section 5.2, and the ensemble clustering algorithm is also SE [22].
- *CCSS-SE*: the basic partition clustering algorithm “CCSS” is our fast CSC algorithm (Algorithm 1), and the ensemble clustering algorithm is also SE [22].
- *CCSS-SER*: the basic partition clustering algorithm is CCSS, and the ensemble clustering algorithm “SER” is our spectral ensemble clustering with random projection (Algorithm 2).

In the phase of basic partition clustering, we fix the number of basic partitions as 50 and the parameters of basic clustering algorithms are the same as those in the last subsection. In addition, similar to the operation of SE [22], the basic partitions are obtained by varying the cluster number from *k* − 5 to *k* + 4. We repeat each cluster ensemble algorithm 10 times and present the average values of results.

**Figure 3:**
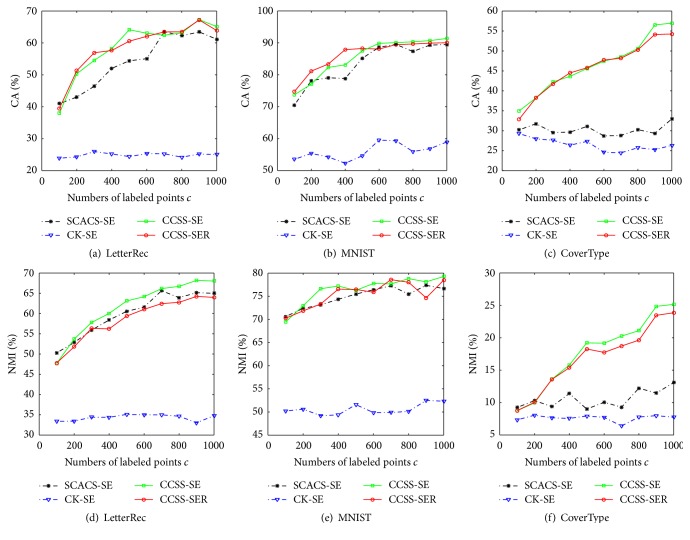
Performance of ensemble clustering algorithms with different constraint information.

First, we show the comparison of different cluster ensemble algorithms in terms of different constraint information in Figure 3. Here the dimensionality *rd* of CCSS-SER reduced by random projection is 40 and we change the number of labeled points *c* from 100 to 1000 with step size 100. In the figure, the validity measures of Figures 3(a)–3(c) and Figures 3(d)–3(f) are related to CA and NMI, respectively. Just like the results of last subsection, CCSS-SE has similar performance to that of SCACS-SE on LetterRec and MNIST data sets and has much better performance on CoverType data set. From the comparison between Figure 1 and 3, we can see that the two validity measures are both higher than those of the basic partition dramatically, verifying ensemble clustering's improvement in clustering quality. Compared with CK-SE, CCSS-SE and CCSS-SER both have better performance significantly, which indicates that the basic partitions have an obvious impact on the final result and also verify the high accuracy of our new constrained spectral cluster ensemble method. In addition, the little difference of performance between CCSS-SE and CCSS-SER implies that the random projection can preserve the results of spectral ensemble clustering approximately on different constraint information.

**Figure 4:**
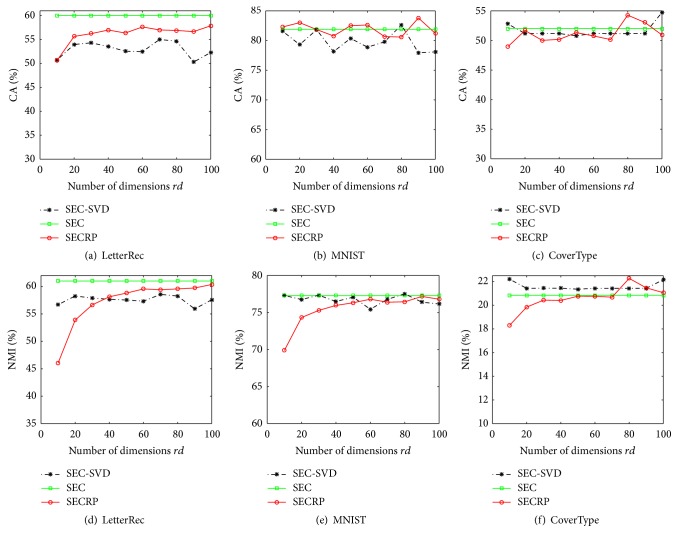
Performance of ensemble clustering algorithms with different dimension.

Second, we inspect the influence of dimensions of random projection on the performance of our algorithm in Figure 4 and Table 2. In Figure 4, the “SEC-SVD” denotes the SEC algorithm with dimensionality reduction of SVD. When *rd* is above certain bound, the validity measures of “SECRP” (denote our algorithm SECRP) are almost stable and similar to those of SEC over all three data sets. This indicates that the accuracy of clustering algorithm can be kept when the dimensions surpass a certain bound, which verifies Theorem 7. The small bound of dimensions (*rd* = 40) also reveals the effectiveness of dimensionality reduction of random projection. With respect to SEC-SVD, although it can also preserve the accuracy of clustering algorithm, its running time is not encouraging. Even letting *rd* = 20, the running time comparisons of original algorithm and SVD method over three data sets are 3.47 s/10.85 s, 4.91 s/14.54 s, and 22.06 s/326.61 s. These phenomena may be caused by the tardiness of SVD on large matrix and the breaking of sparseness of binary matrix **B**. In Table 2, the decrease of running time verifies the efficiency of our new spectral ensemble clustering. Combining this and subfigures (g,h,i) in Figure 1, the efficiency of new constrained cluster ensemble method is also verified. In addition, we can see the decrease of running time caused by random projection is declining with the growth of dimensions, indicating the relative small dimensionality with random projection is preferable.

## 6. Conclusion

To handle large scale data sets, we propose a fast CSC algorithm. The new algorithm can decrease the space and time complexity of a recently introduced CSC model through landmark-based graph construction and improve its efficiency further by random sampling. The new algorithm not only has the similar property of original model asymptotically, but also is the most efficient and suitable to a wide range of data sets empirically. Taking the new CSC algorithm as basic partition algorithm, we design an efficient semisupervised cluster ensemble algorithm. In the stage of consensus clustering, we reduce the dimensionality of input of spectral ensemble clustering by sparse random projection and prove that the sparse random projection can keep the clustering quality approximately. The experimental results over several data sets also verify the efficiency and effectiveness of new cluster ensemble algorithm. Moreover, in the process of spectral ensemble clustering, the influence analysis of dimensionality reduction with random projection can also give the theoretical guarantee for the weighted *k*-means clustering with random projection. In the future, we will use techniques such as applying several different basic partition methods, selecting the results of basic partitions, and giving different weights for basic partitions to improve the performance of our cluster ensemble algorithm further.

## Figures and Tables

**Algorithm 1:**
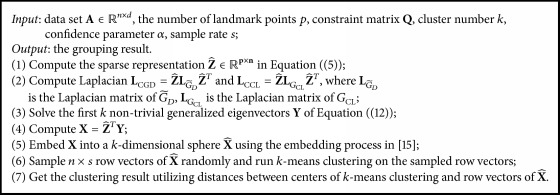
Fast constrained spectral clustering.

**Algorithm 2:**
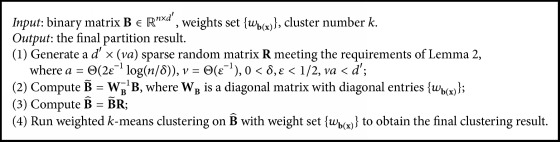
Spectral ensemble clustering with random projection.

**Table 1:** Data sets information.

Data set	#instances	#attributes	#classes
Letter recognition	20,000	16	26
MNIST	70,000	784	10
CoverType	581,012	54	7

**Table 2:** Decrease of running time of SECRP from SEC with different dimensions *rd*.

*rd*	10	20	30	40	50	60	70	80	90	100
LetterRec	2.44	2.39	2.11	2.07	2.04	2.03	1.92	1.74	1.67	1.56
MNIST	2.76	2.68	2.66	2.58	2.51	2.34	2.31	2.11	2.16	2.03
CoverType	18.85	18.64	15.34	15.26	14.04	11.43	9.73	8.31	7.72	7.44
